# Efficacy and Safety of Brexpiprazole in Agitation Associated with Dementia in Alzheimer's Disease: A Systematic Review and Meta‐analysis

**DOI:** 10.1002/alz70858_103458

**Published:** 2025-12-26

**Authors:** Hammad Javaid, Muhammad Nabeel Saddique, Ayaan Musani, Anurag Jha, Meeram Noor, Mahnoor Arfan, Umaima Cheema, Maryyam Aqeel, Shamikha Cheema

**Affiliations:** ^1^ King Edward Medical Univseristy, Lahore, Punjab, Pakistan; ^2^ Nibras Research Academy, Lahore, Punjab, Pakistan; ^3^ Wildfrid Laurier University, Waterloo, ON, Canada

## Abstract

**Background:**

Agitation is one of the most distressing neuropsychiatric symptoms in patients with dementia due to Alzheimer's disease (AD), significantly impacting patients' quality of life and increasing caregiver burden. Current management options for agitation in AD remain limited, with many treatments associated with modest efficacy and considerable safety concerns. Brexpiprazole, a serotonin‐dopamine activity modulator, has recently emerged as a potential therapeutic option for this challenging clinical scenario. This systematic review and meta‐analysis aim to evaluate the efficacy and safety of brexpiprazole in managing agitation associated with dementia in AD.

**Method:**

A comprehensive literature search was conducted across PubMed, Cochrane, Scopus, Embase and ClinicalTrials.gov from inception up to January 2025. We pooled dichotomous outcomes as risk ratio (RR) and continuous outcomes as mean differences (MD) with 95% confidence intervals (CI), using random‐effects models. Heterogeneity was assessed using I^2^ and X^2^ statistics. A *p*‐value of <0.05 was considered statistically significant. All the calculations were performed using RevMan 5.4.

**Result:**

This meta‐analysis included 4 studies involving 1440 patients (944 vs. 496) suffering from agitation associated with dementia in AD. Brexpiprazole demonstrated a significant reduction in the Cohen‐Mansfield Agitation Inventory (CMAI) score (MD: ‐3.94 [95% CI: ‐6.21 to ‐1.67], *p* <0.001) and Neuropsychiatric Inventory‐Nursing Home (NPI‐NH) score (MD: ‐0.67 [95% CI: ‐1.08 to ‐0.26], *p* = 0.002) from baseline, with the greatest improvement observed at the 2–3 mg dose. There was statistically significant change in SAS score (MD: 0.38, [95% CI: 0.18 to 0.58], *p* = 0.0002) but no difference in Mini‐Mental State Examination (MMSE) score (MD: ‐0.26 [95% CI: ‐0.53 to 0.01], *p* = 0.06) and Clinical Global Impression‐Severity (CGI‐S) score (MD: ‐0.16 [95% CI: ‐0.34 to 0.01], *p* = 0.06) from the baseline with brexpiprazole. There was no difference between two groups regarding serious adverse events (*p* = 0.57), dizziness (*p* = 0.49), extrapyramidal effects (*p* = 0.18) and mortality (*p* = 0.22).

**Conclusion:**

Brexpiprazole significantly reduces agitation symptoms in Alzheimer's disease, as shown by improvements in CMAI and NPI‐NH scores, with no significant cognitive or safety concerns. While promising, limitations include moderate heterogeneity and short follow‐up durations. Future studies should focus on long‐term effects, quality of life, and identifying optimal patient subgroups for treatment.

